# Mapping loss of heterozygosity in normal human breast cells from BRCA1/2 carriers

**DOI:** 10.1038/sj.bjc.6603298

**Published:** 2006-08-01

**Authors:** C L Clarke, J Sandle, A A Jones, A Sofronis, N R Patani, S R Lakhani

**Affiliations:** 1 Breakthrough Toby Robins Breast Cancer Research Centre, Institute of Cancer Research, London SW3 6JB, UK; 2 Department of Surgery, University College London, Charles Bell House, 67-73 Riding House Street, London W1W 7EJ, UK

**Keywords:** normal breast, loss of heterozygosity, BRCA1, BRCA2

## Abstract

We have studied loss of heterozygosity at the BRCA1 and BRCA2 loci in 992 normal cell clones derived from topographically defined areas of normal tissue in four samples from BRCA1/BRCA2 mutation carriers. The frequency of loss of heterozygosity in the clones was low (1.01%), but it was found in all four samples, whether or not a tumour was present. Topographical mapping revealed that the genetic changes were clustered in some breast samples. Our study confirms the previous finding that a field of genetic instability can exist around a tumour, suggesting that sufficient tissue must be removed at surgery to avoid local recurrence. We also demonstrate that such a field of genetic change can exist in morphologically normal tissue before a tumour develops and, for the first time, we demonstrate that the field is of a size greater than one terminal duct-lobular unit. The genetic changes are not identical, however, which suggests that genetic instability in these regions may play an early role in tumour development. We also confirm and extend our original observation of loss of the wild-type BRCA1 allele in some clones, and loss of the mutant allele in others, demonstrating that loss of either allele is a stochastic event.

The term breast cancer encompasses a variety of tumour types with differing clinical behaviour. In an attempt to understand the pathways that give rise to these tumours, a number of studies have examined the genetic changes in precursor lesions. Loss of heterozygosity (LOH) studies have shown genetic alterations in ductal carcinoma *in situ* (DCIS) (Stratton *et al*, 1995; Farabegoli *et al*, 2002), lobular carcinoma *in situ* (Lakhani *et al*, 1995a; Vos *et al*, 1997) atypical ductal hyperplasia (Lakhani *et al*, 1995b) and nonatypical hyperplasia of the breast (Lakhani *et al*, 1996). Further studies, using comparative genomic hybridisation have also demonstrated a number of genetic changes in DCIS (Buerger *et al*, 1999a, 1999b), LCIS (Lu *et al*, 1998; Buerger *et al*, 2000), hyperplasia of usual type (Jones *et al*, 2003), apocrine hyperplasia (Jones *et al*, 2001) and columnar cell lesions (Simpson *et al*, 2005a). These studies support the idea that there may be several pathways in the multistep model of carcinogenesis (Simpson *et al*, 2005b) and indicate that some benign lesions may be precursors to invasive carcinoma.

It follows that the earliest genetic changes that fit into this multistep model might be detected in cells that appear morphologically normal. Such changes were demonstrated by Deng *et al* (1996) who identified LOH in normal breast lobules adjacent to, but not distant from tumours in sporadic breast cancers. This study was carried out by examining LOH in microdissected morphologically normal lobules. In order for such changes to be detected using these techniques, the LOH must be present in a high proportion of the cells in the microdissected area. These results suggest, therefore, that the LOH probably occurred in a cell that gave rise to the part of lobule that was microdissected; however, it does not distinguish between luminal and myoepithelial cell involvement. Meng *et al* (2004) also demonstrated LOH in microdissected normal lobules adjacent to tumours, and they reported for the first time LOH in the BRCA1, BRCA2 and ATM genes in normal lobules adjacent to sporadic tumours.

We have previously shown that genetic alterations are present in normal breast cells both close to and distant from a tumour and in tissue in which no tumour is present (Lakhani *et al*, 1999). These results were achieved by cloning individual cells from pieces of fresh human breast to give sufficient DNA to examine changes at the single cell level, revealing alterations that might be missed by looking at microdissected tissue. Furthermore, it allowed us to look independently at the luminal epithelial and myoepithelial cells. We established that LOH occurs in both luminal and myoepithelial cells and, in one case, the same LOH was detected in each cell type suggesting that the two cell types came from a common precursor.

Microdissection has been used to demonstrate LOH in normal lobules in familial breast cancers (Cavalli *et al*, 2004). Cavalli *et al* detected LOH in normal tissues adjacent to and, in one case, at a distance up to 8.7 mm from the tumour. In addition LOH was detected in areas of sclerosing adenosis both in the tumour-containing breast and in the contralateral prophylactic mastectomy. In all cases of LOH at the BRCA loci the wild-type allele was lost.

Larson *et al* (2005) compared the levels of LOH in normal lobules from BRCA1 carriers, patients with sporadic cancer and reduction mammoplasty specimens. They found a three-fold increase in the level of allelic imbalance in the normal tissues from patients with sporadic cancers or those with a BRCA1 mutation compared to reduction mammoplasty specimens; however, the distance of the microdissected terminal duct-lobular units (TDLUs) from the tumour was not recorded. Ellsworth *et al* (2004) examined the distribution of LOH in microdissected samples from different quadrants of mastectomy specimens in sporadic breast cancer. They demonstrated increased levels of LOH in the outer quadrants of the breast, with the highest level in the lower outer quadrant in which the majority of tumours were found.

In the current study we have applied our cell cloning technique to mastectomy and prophylactic mastectomy samples from patients with a mutation in either BRCA1 or BRCA2. We have analysed the frequency of LOH in these samples, the losses of mutant and wild-type alleles, and by taking tissue from defined areas of the breast we have mapped the distribution of the losses.

## MATERIALS AND METHODS

### Tissue samples

The use of normal breast tissue was approved by the Ethics Committee of the Royal Marsden Hospital and the Institute of Cancer Research. Normal breast tissue samples were obtained with informed consent from three patients undergoing mastectomy and/or prophylactic mastectomy. The first case consisted of a tumour-bearing (mastectomy) specimen. In the second case, the whole mastectomy specimen was required for pathological diagnosis; however, samples from the contralateral prophylactic mastectomy were available for this study. In the third case, tissue was available from both the tumour-carrying breast and the contralateral unaffected breast. In total, therefore, we used a total of four separate breast specimens: two mastectomies and two prophylactic mastectomies. Fresh unfixed breast specimens were sliced and examined by a pathologist and pieces of normal tissues from multiple sites throughout the breast were selected and their positions recorded. The rest of the specimen was examined and processed routinely. Paraffin-embedded samples of tumour-free lymph node removed at surgery were used as normal controls. Paraffin embedded tumour samples were used to investigate LOH in the tumour and, by inference, establish which were the mutant and wild-type alleles, the loss in the tumour being assumed to be wild-type.

### Normal cell cloning

Tissue samples were cut into small fragments using scissors and/or scalpels and then incubated with stirring at 37°C overnight in a solution of 0.5 mg ml^−1^ collagenase (Type 1, Sigma, Poole, Dorset, UK) in Leibovitz L-15 medium plus 5% foetal calf serum. The tissue fragments were collected by centrifugation and then passed sequentially through a 70 *μ*m and a 40-*μ*m cell strainer (Falcon) to collect the epithelial fragments and remove the single cells consisting predominantly of fibroblasts, endothelial and blood cells. The epithelial fragments were plated in RPMI medium plus 10% FCS, 5 *μ*g ml^−1^ hydrocortisone, 5 *μ*g ml^−1^ insulin and 100 ng ml^−1^ cholera toxin (all additives from Sigma). The epithelial cells were allowed to mobilise from the fragments for approximately 5 days, and were then trypsinised to form a single-cell suspension. The cells were plated in 15 cm Petri dishes (1000 cells per dish), which had been preseeded with 10^6^ lethally irradiated (40 Gy) mouse 3T6 fibroblasts. The clones were grown in Ham's F12 medium plus 10% FCS, 1 *μ*g ml^−1^ hydrocortisone, 5 *μ*g ml^−1^ insulin, 10 ng ml^−1^ human recombinant EGF and 100 ng ml^−1^ cholera toxin (all additives from Sigma). This medium results in the clonal growth of both luminal and myoepithelial cells, but does not allow the growth of primary tumour cells under clonal conditions (Wolman *et al*, 1985; O'Hare, 1990). After 10–14 days the clones of epithelial cells were fixed for 3 min in ice-cold methanol, rinsed in several changes of PBS and stored in PBS for immunofluorescent labelling.

### Immunofluorescent labelling

The clones were labelled by triple immunofluorescence for the expression of cytokeratins 18 and 19 (predominantly luminal cells *in vivo*) and cytokeratin 14 (predominantly myoepithelial cells *in vivo*). Two antibodies were used to identify luminal cell clones because, in our hands, up to 30% of luminal clones will have only weak or absent expression of one or other of the cytokeratins in culture (unpublished observation). The primary antibodies were applied as a cocktail as follows: LL002 (mouse monoclonal IgG3 anti-CK14, Novocastra) 1:200, DC10 (mouse monoclonal IgG1 anti-CK18, Novocastra) 1:200, LP2K (mouse monoclonal IgG2b anti-CK19, a kind gift of Professor EB Lane, Dundee, UK) 1 : 5. These antibodies were detected with a cocktail of goat-anti-mouse subclass-specific secondary antibodies (Southern Biotechnology) as follows: biotinylated anti-IgG3, FITC anti-IgG1, Texas Red anti-IgG2b, followed by a tertiary layer of streptavidin AMCA, resulting in CK18 seen as green, CK19 blue and CK14 red. The clones were examined using a Zeiss Axiovert fluorescence microscope and individually scored for the expression of each antigen to identify the cell type of origin.

### LOH analysis

Following identification of cell type by immunofluorescence, each clone was scraped from the culture plate. DNA was extracted from the clones or the paraffin sections of tumour or normal lymph node control using proteinase K digestion as previously described (Lakhani *et al*, 1996). Dinucleotide repeat regions of polymorphic microsatellite markers ranging in size from 119 to 185 base pairs in the regions of the BRCA1 or BRCA2 genes were examined. These markers were: D17s250, D17s800, D17s806 and D17s1814 in the region of BRCA1 (17q21), D17s855, D17s1323 and D17s1322 intragenic of BRCA1, and D13s267, D13s260 and D13s1293 in the region of BRCA2 (13q13). Loss of heterozygosity was investigated at these markers by amplification using fluorescently tagged primers and the polymerase chain reaction (PCR). The products were run on an ABI prism 377 and the data were processed using GeneScan Analysis software (version 3.1) followed by Genotyper software (version 2.5).

## RESULTS

### LOH frequency and distribution

Clones from four breast samples (one from a BRCA2 mutation carrier, three from BRCA1 mutation carriers) were analysed for the presence of LOH. Of these, two samples had tumour present and two were prophylactic mastectomies. For the prophylactic mastectomies, in each case the contralateral breast contained tumour. A summary of the results of these analyses are presented in Table 1 including the number of clones harvested from each breast sample, and the number of microsatellite markers examined per sample. In the BRCA2 sample, all three microsatellites studied were informative, but no further DNA was available to study other markers. In the BRCA1 samples, seven microsatellite markers were studied (six BRCA1 and one BRCA2), but only six were informative in each case: D17s800 was not informative (homozygous) in sample 2, and D17s1322 was not informative in samples 3 and 4. In total, 5355 microsatellites were examined across the four samples. In these samples the wild-type allele was considered to be the allele lost in the tumour samples.

**Figure 1:**
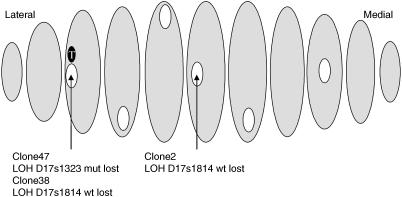
Distribution of LOH detected in areas of histologically normal tissue of a mastectomy specimen (sample 3). Areas of tissue from which cells were cloned are shown in white, tumour is shown in black (T). Wt=loss from wild type allele, mut=loss from mutant allele.

**Figure 2:**
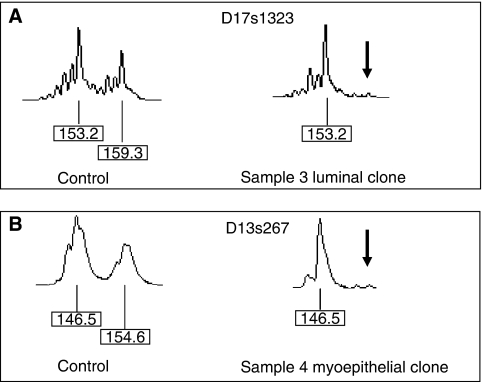
Loss of heterozygosity in normal cell clones demonstrated by amplification of microsatellites D17s1323 (**A**) and D13s267 (**B**). The normal tissue control for each sample shows two allele peaks. The ‘normal’ clones from samples 3 and 4 each demonstrate loss of one allele (arrow).

The rate of LOH in each case was low (0.739–1.579% of clones) but it was detected in all samples, both in the presence and absence of tumour. In sample 1, two luminal clones had LOH at D13s1293, both losses of the peak from the wild-type allele (hereafter referred to as loss of wild-type). In sample 2, one luminal and one myoepithelial clone showed LOH at D17s806 (loss of wild-type). Sample 3 had a total of three LOH all in luminal epithelial cells. Two of the clones were adjacent to the tumour, one showing LOH in D17s1814 (loss of wild-type) and the other in D17s1323 (loss of mutant). The third clone with LOH at D17s1814 (loss of wild-type) appears to be distant from the tumour and the other clones showing LOH (Figure 1), but it is in fact within 2 cm of the other alterations since the breast tissue was cut into very thin slices. Sample 4 (the contralateral breast to sample 3) also had three clones within a small area of breast exhibiting LOH (two in the same slice, one within 1 cm). The cell type and alterations were, however, all different: one luminal clone had LOH at D17s1814 (loss of wild-type), one myoepithelial clone had LOH at D17s1323 (loss of mutant) and another had a loss at D13s267 (a BRCA2 microsatellite). In each case, no clone had LOH at more than one microsatellite. Examples of LOH found in clones from samples 3 and 4 are shown in Figure 2.

### Wild-type and mutant alleles

A summary of the losses from the wild-type and mutant alleles is presented in Table 1. A total of nine LOH were detected in the regions of BRCA2 in sample 1 and BRCA1 in samples 2–4. Of the nine LOH, seven were wild-type and two mutant losses. Two of the wild-type losses were in clones from the BRCA2 case; however, D13s267 which is closer to BRCA2 showed no LOH, and therefore the gene was not likely to be inactivated. In the BRCA1 cases, both of the mutant losses were intragenic, and the wild-type losses were in the markers closest to the BRCA1 gene. In the latter cases, it was not clear whether the area of loss extended into the gene, and since there was not enough DNA available to sequence the gene we could not establish whether the wild-type allele was inactivated. The proportions of losses of microsatellites on the wild-type and mutant alleles were tested to see if they were equal using a *χ*^2^ test, and the difference did not reach significance (*P*=0.22).

## DISCUSSION

In this study we have investigated the frequency and distribution of LOH in normal breast tissue from patients carrying a mutation in the BRCA1 or BRCA2 genes, using a single cell cloning technique.

It might be argued that the cloning process may produce LOH, particularly in cells with BRCA1/2 mutations. If the alterations occurred during the growth of the clone, however, only a proportion of the cells would carry the LOH, and this would be reflected in the levels of each peak on the Genescan analysis. In this study, each clone showed complete loss of one peak, indicating that the LOH was present in all the cells, and therefore must have occurred before the growth of the clone.

In our previous study only one LOH was found in three reduction mammoplasty samples (Lakhani *et al*, 1999). In contrast, both of the prophylactic mastectomies (from BRCA1/2 mutation carriers) in the current study contained clones with LOH. This result is consistent with the previously reported three-fold increase in LOH in normal TDLUs from BRCA1 carriers compared to reduction mammoplasties (Larson *et al*, 2005). The level of LOH in the mastectomies (with tumour) from the two studies, however, appears to show a large difference: 0.83% of clones in the BRCA cases and 7.6% in the non-BRCA cases. The majority of LOH found in the non-BRCA cases, however, were in a single sample in which all clones showed LOH, suggesting that the loss occurred very early in the development of the breast. Without this case, the level of LOH in the non-BRCA cases drops to 0.68%. This result illustrates the difficulty of examining changes that could potentially occur at any time during the development of the breast and thus affect anything from a single cell to the whole organ.

These studies are particularly labour intensive, making it impractical to examine many samples in order to produce statistically significant results. Nevertheless, some interesting observations of distribution and allele loss have still arisen from this study. We have shown that LOH is found close to tumours, and is sometimes found in other areas of the breast. In particular, we have shown that genetic alterations at the single-cell level may be clustered, and extend to an area that consists of more than one TDLU. Such clustering suggests that a change may have occurred in a precursor cell, which gave rise to that area of the breast. This result complements the findings of Tot (2005), who observed that multiple foci of DCIS are sometimes found within a single lobe of the breast, and suggests that an early genetic event may give rise to a ‘sick lobe’. In our samples, it is unlikely that an early event in BRCA1 is responsible for the apparent field of genetic instability since the LOH vary in the different clones in a single area. Indeed, it is not clear whether the losses from the wild-type allele in the clones from the BRCA1 cases extend into the gene, and thus we cannot comment on whether BRCA1 function is lost. Although this clearly has implications for disease pathogenesis since losses that do not extend into the BRCA1 gene should not contribute to breast tumourogenesis, nevertheless, the clustering of the losses still suggests the presence of an underlying instability in an area of the breast. In order to further elucidate the mechanism of genetic change in these areas, it would be interesting to analyse the whole genome using SNP arrays (Zhou *et al*, 2005), and we anticipate that this approach will become possible as DNA amplification methods improve.

Our finding of clusters of LOH has clinical implications. Firstly, our results may help to account for some local tumour recurrences following apparent surgical clearance. The ‘recurrence’ may actually be a new tumour formed in the area of underlying genetic changes close to the original tumour. If this is the case, it implies that the rate of ‘recurrence’ may be reduced by removing more tissue at surgery. Secondly, one patient had two clusters of LOH, one close to the tumour and the other in the contralateral breast. It is impossible to know whether the area of LOH in the non-diseased breast might have gone on to form a clonal proliferation/tumour.

Our studies have demonstrated a second novel observation: loss of either the mutant or wild-type allele at microsatellite markers in the BRCA1 region (Lakhani *et al*, 1999). Loss of the wild-type BRCA1 or BRCA2 allele has been shown in breast tumours arising in patients carrying a germline mutation in one of these genes (Collins *et al*, 1995; Cornelis *et al*, 1995; Merajver *et al*, 1995; Osorio *et al*, 2002) and in some sporadic breast tumours (Hanby *et al*, 2000; Katsama *et al*, 2000; Johnson *et al*, 2002). The loss of the wild-type allele is presumed to be one of the first genetic events leading to tumour formation in familial breast cancer cases (Cornelis *et al*, 1995). It can be argued that the mutant allele is equally likely to be lost in normal cells, but since this will result in the maintenance of haploid sufficiency of the BRCA genes, the cells should not be expected to go on to form a tumour. Our results show that microsatellite markers on either the wild type or the mutant allele may be lost; however, Cavalli *et al* demonstrated only loss of the wild-type allele in their normal breast samples (Cavalli *et al*, 2004). The difference in the results suggests that although LOH at either allele may be a stochastic event, it appears that only those cells that have lost the wild-type allele go on to form clonal expansions that will populate the lobules. It is possible that the loss of functional BRCA1 may lead to a series of genetic alterations that give the cells a growth advantage so that they are more likely to form areas carrying the LOH. Interestingly, in our previously reported sample (Lakhani *et al*, 1999), one of the clones with a confirmed intragenic wild-type loss also showed losses at 11p and 13q suggesting that this process may have been underway.

We have shown in this study that areas of genetic alterations are present in normal breast tissues of BRCA1/2 carriers, and that LOH at BRCA1/2 is possibly an early, but not necessarily the earliest event. Further studies using alternative techniques such as SNP arrays will be required to fully understand the sequence of events that leads to tumour formation in these tissues.

## Figures and Tables

**Table 1:** The number of LOHs detected in samples of ‘normal’ human breast tissue from two mastectomies and two prophylactic mastectomies

**Sample no.**	**Specimen**	**No. of clones**	**No. of informative microsatellite markers**	**No. of LOH**	**% of clones with LOH**
1	Mastectomy BRCA2	199	3	2 wt	1.005
2	Prophylactic BRCA1	197	6	2 wt	1.015
3	Mastectomy BRCA1	406	6	2 wt, 1 mut	0.739
4	Prophylactic BRCA1 (contralateral to sample 3)	190	6	1 wt, 1 mut, 1 non-BRCA1	1.579

Wt=loss from wild-type allele, mut=loss from mutant allele.
